# Association of plasminogen activator inhibitor-1 and angiotensin converting enzyme polymorphisms with recurrent pregnancy loss in Iranian women

**Published:** 2015-10

**Authors:** Fatemeh Shakarami, Mohammad Taghi Akbari, Shohreh Zare Karizi

**Affiliations:** 1 *Department of Biology, Guilan University, Rasht, Iran.*; 2 *Department of Medical Genetics, Faculty of Medical Sciences, Tarbiat Modares University, Tehran, Iran.*; 3 *Tehran Medical Genetics Laboratory, Tehran, Iran.*; 4 *Department of Biology, Varamin Pishva Branch, Islamic Azad University, Varamin Pishva, Iran.*

**Keywords:** Spontaneous abortion, Thrombophilia, Plasminogen activator inhibitor-1, Angiotensin converting enzyme

## Abstract

**Background:**

Recurrent pregnancy loss (RPL) defined by two or more failed pregnancies before 20 weeks of gestation. Several factors play a role in RPL including thrombophilic conditions which can be influenced by gene polymorphisms. Plasminogen activator inhibitor-1 (PAI-1) and angiotensin converting enzyme (ACE) genes are closely related to fibrinolytic process, embryonic development and pregnancy success.

**Objective:**

The aim of this study was to investigate the relationship between RPL and common polymorphisms in ACE and PAI-1 genes.

**Materials and Methods:**

In this case control study, 100 women with recurrent abortions (at least two) were selected as cases and 100 healthy women with two or more normal term deliveries without a history of abortion as controls. Total genomic DNA was isolated from blood leukocytes. The status of the PAI-1 4G/5G and ACE (D/I) polymorphism was determined by PCR-RFLP.

**Results:**

Homozygosity for PAI-1 4G polymorphism was seen in 17 cases (17%), and 5 controls (5%) (p=0.006) so patients with homozygote 4G mutation were significantly more prone to RPL in contrast to control group (OR: 4.63, % 95 CI: 1.55-13.84). In addition, 7 patients (7 %), and no one from the control group, were homozygote (I/I) for ACE polymorphism (p=0.034), suggesting no significant associations between ACE D allele or DD genotype and RPL.

**Conclusion:**

Considering these results, because 4G/4G polymorphism for PAI-1 gene could be a thrombophilic variant leading to abortion, analysis of this mutation and other susceptibility factors are recommended in patients with RPL.

## Introduction

Abortion is one of the most common pregnancy complications and is referred to pregnancy loss before the 20th week of gestation with various maternal and fetal reasons. Recurrent pregnancy loss (RPL) means occurring two or three successive recurrent abortions before the 20th week of gestation (1, 2). Fifty percent of RPL are caused by anatomical, immunological, genetics, endocrine, thrombophilic and environmental factors. However, in 50% of the cases, the cause of abortion is unknown or idiopathic (3). Since more than half of all the cases of idiopathic RPL are considered to be associated with thrombophilia disorders, analysis of polymorphisms of effective genes in thrombophilia is thought logical. PAI-1 plays an important role as a glycoprotein (Molecular weight: 50kDa) in inhibiting fibrinolysis reactions. Conversion of plasminogen to plasmin as an important milestone in the fibrinolytic is set by such activators as tissue plasminogen (t-PA) and urokinase plasminogen (u-PA) (4). The PAI-1 is the fastest t-PA inhibitor and regulates the rate of clot formation in the coagulation system. Recently, it has been proved that plasma and placental concentration of PAI-1 in women who had a history of preeclampsia is more than those in fertile healthy women, and an increase in plasma level of PAI-1 leads to a decrease in fibrinolysis activities (4). Angiotensin II increases plasma level of *PAI-*1. Angiotensin converting enzyme (ACE) is a copper protease that affects converting Angiotensin I into Angiotensin II. The latter is involved in the synthesis of PAI-1 by controlling the coagulation process (4). PAI-1 gene has 9 exons and its length is approximately 12 kb and is located on chromosome 7q21.3-q22. The common polymorphism in the PAI-1 gene promoter region is clearly associated with variation in its level. This polymorphism is a change in the five consecutive guanosine in the 675 bp upstream from the start site of transcription, and its conversion to four guanosines, leads to increased production of PAI-1 in response to various stimuli (5). Homozygous individuals for 4G alleles have higher level of PAI-1 in plasma, whereas heterozygous and homozygous individuals for 5G allele have lower level of PAI-1. Human ACE gene has 26 exons with the length of 21 kb and is located on chromosome17q23.3. There are several polymorphisms in the ACE gene that its most common form is the insertion or deletion (I/D) of a 278 bp fragment in intron 16 of the gene that regulates the amount of the enzyme (6). Polymorphism of the ACE I/D is significantly correlated with the level of ACE enzyme circulation. Average level of ACE plasma in individuals with D/D genotype is approximately two times more than those with I/I genotype. However, those with I/D genotype have the average level of enzyme circulation (5). Several studies have been conducted on the relationship between PAI-1 and ACE gene polymorphisms and RPL and in different populations conflicting results have been obtained from these studies. The aim of this study was to investigate the association of PAI-1, 4G/5G (rs1799889) and ACE I/D polymorphism with RPL in Iranian patients. 

## Materials and methods

**Sampling procedure**

In this case-control study, 100 patients with the history of at least two spontaneous abortions were selected as the case group from the cases with RPL referred to Tehran Medical Genetics Laboratory during years April 2013 and November 2014. These patients were examined in terms of known factors affecting RPL including: anatomical abnormalities, immunological test for autoantibodies, the factors associated with the endocrine, and they were proved to be normal with regard to all those factors. All patients had negative TORCH and pap-smear tests results. 100 women with at least two successful births, with no history of abortion, endometriosis or infertility were recruited as the control group (Table I). Written consent was taken from all of the participants in both groups after being informed of the purpose of the research. 

**Genotype determination**

In this study, 5 ml peripheral blood from all the participants was collected in EDTA tubes and DNA was extracted by standard salting out method (8). Genomic DNA was amplified by polymerase chain reaction (PCR) using gene specific primers (Table II). PCR reaction for PAI-1 and *ACE* gene was performed with the final volume of 25 µl containing 80 ng of genomic DNA, 1X PCR Buffer, 0.25 mM of each dNTPs, 1.5 mM of MgCl2, 5-7 picomoles of each primer, and 0.5 unit of Taq DNA polymerase (Sinagen, Iran). PCR conditions were: 95^°^C for 5min, following by 95^°^C for 50s, 69^°^C for 50s, 72^°^C for 50s for 30 cycles and finally 72^°^C for 10min. The main PCR product of PAI-1 gene that is 148bp in length has no cutting site for BseRI enzyme (New England Biolabs). However, changes in the promoter region of five consecutive guanosines and their conversion into four guanosines create the enzyme cleavage site and cleave the amplicon into two fragments (110 bp and 38 bp). The ACE D/I genotype was characterized by the length of the PCR product, 190 bp in the case of the deletion and 490 bp in the presence of the insertion (9). The resulting fragments were electrophoresis on 12% polyacrylamide gel.

**Statistical analysis**

The allele and genotype frequencies for each polymorphism in both case and control groups were determined by Chi-square and p<0.05 was considered as significant and odd ratio was calculated for each group.

## Results

According to the results observed by electrophoresis of PCR and RFLP product, the genotype of every individual for 4G/5G polymorphism of PAI-1 gene and D/I polymorphism of ACE gene were determined. The results of the allele and genotype frequency, the differences in genotype distribution, and relative risk for each polymorphism are summarized in tables I and II. In the present study, the frequency of 4G allele in women with RPL was 42% and in the control group was 30%, which showed that the 4G allele frequency in the patient group increased significantly (p=0.012) compared to the control group, and its relative risk for the population was approximately 1.69. The case is also true for the 4G/4G genotype with the frequency of 17% in the patient group and 5% in the control group, (p=0.006) and its relative risk of 4.63. 

The frequency of ACE I allele in the patient and control group was 36.6% and 24%, respectively. In fact, contrary to what was expected, I allele despite being the normal allele showed significantly higher frequency in the patient group compared to the control group (p=0.007). 

The I/I genotype also showed a higher frequency (6%) in the patient group as compared to the control group who lacked this genotype (p=0.034).

**Table I:** polymerase chain reaction primer sequences for ACE and PAI-1 polymorphisms

**Polymorphism**	**Primers**	**PCR product**
ACE intron 16 I/D	F-5′CTGGAGACCACTCCCATCCTTTCT3 R-5′GATGTGGCCATCACATTCGTCAGAT3	Depends on genotype
PAI-1 -675 4G/5G	F-5′CACAGAGAGAGTCTGGACACGTGA3′ R-5′TGCAGCCAGCCACGTGATTGTCTAG3	148 bp (110, 38)

PCR: polymerase chain reaction

**Table II:** PAI-1 (4G/5G) genotype and allele frequencies for case and control group

	**Controls ** **n (%)**	**Cases ** **n (%)**	**OR (**95 % CI**)**	**p-value**
**Allele**				
5G	140)70)	116)58)	1	-
4G	60)30)	84)42)	1.69 (1.11, 2.55)	0.0127
**Genotype**				
5G/5G	45)45)	33)33)	1	-
5G/4G	50)50)	50)50)	1.36 (0.75, 2.47)	0.31
4G/4G	5)5)	17)17)	4.63 )1.55, 13.84)	0.006

Genotype and allele frequencies between patients and control subjects were compared by Pearson’s chi-squared test. p<0.05 was considered statistically significant

**Table III:** Angiotensin converting enzyme (D/I) genotype and allele frequencies for case and control group

	**Controls n (%)**	**Cases n (%)**	**OR (**95 % CI**)**	p-value*
D	152)76)	128)63.3)	1	-
I	48)24)	74)36.6)	1.82 (1.18, 2.8)	0.007
				
D/D	52)52)	34)34)	1	-
D/I	48)48)	60)60)	1.68 (1.57, 9.63)	0.031
I/I	0)0)	6)6)	12.11 (1.7, 36.9)	0.034

Genotype and allele frequencies between patients and control subjects were compared by Pearson’s chi-squared test. p<0.05 was considered statistically significant

## Discussion

Among women suffering from RPL, in almost 50% of the cases, the cause of abortion is unknown. Several studies suggest that thrombosis in placental microvasculature might be the reason for the idiopathic abortions. According to the previous studies, there are evidences of relationships between PAI-1 and ACE genotypes and increased risk of all kinds of thrombophilic conditions such as recurrent abortions (10-20). Thus the study of these polymorphisms in women with idiopathic RPL could shed light on their association with RPL. In the present study, the frequency of 4G allele and 4G/4G genotype in patients group is significantly increased. This finding is in line with two previous studies in Iran by Soltangharaee (2007) and Arabi (2011) (11, 12). 

This significant association has also been observed in the study of Goodman (2009) in the US, Guan (2005) in China, and Al Sallout (2011) in Gaza (13, 14). In contrast, other findings including the findings of Dossenbach-Glaning (2003) in Austria, Ivanov (2011) in Bulgaria, and two studies conducted by Coulam (2006, 2008) in the US do not show significant association between 4G/4G genotypes and RPL (10, 15-17). So, there are contradictions in results of studies about PAI-1 4G/5G polymorphism and recurrent abortions, even in the studies performed in the same country. An example of these contradictions is the study of Wolf (2003) in Germany. They evaluated association between fibrinolysis factors and RPL, and reported that 51% of patients and 49% of controls group for 4G variant in PAI-1 gene were heterozygotes and 35% of patients and 31% of controls group were homozygous for this polymorphism. Therefore, frequency of both allele 4G and genotype 4G/4G were almost similar between the case and control groups (18). On the contrary, in another study conducted in Germany by Schenk (2008), the frequency of 4G alleles (4G/4G or 4G/5G genotypes) was significantly higher in the patients group compared to the controls group (19). In the present study, there were no evidence that significant association existed between D/D genotype and D allele of the ACE gene comparing the patients and control group with regard to the role of ACE polymorphism in RPL.

While this relationship has been observed in Caucasian patients (9). The systematic review and meta-analysis conducted by Su (2013) investigating the association between ACE polymorphism and recurrent abortions, where 11 eligible studies including 1275 patients and 2049 controls were analyzed, assuming dominant inheritance model and a significant association was identified. They also showed a significant association between polymorphism of the ACE I/D with recurrent pregnancy loss and showed that women with I/D and D/D genotypes had higher risk (1.29 OR) than women with I/I genotype (20). These inconsistent results are probably due to differences in populations studied, their genetics backgrounds (genetics drift, population migration or natural selection) and the sample size.

Biological functions of PAI-1 and ACE are important in the regulation of fibrinolytic activity in placentation and trophoblast invasion during human pregnancy. The results of the present study showed that 4G allele and 4G/4G genotypes are contributing factors in PRL. Considering the results of this study and the previous studies conducted in Iran with regard to the relationship between these factors and the pathogenesis of recurrent pregnancy loss and fetal factors associated with recurrent abortion, the hypothesis of the relevance of this factor with the RPL is still unanswered. Therefore, to investigate this factor and its association with RPL in detail, further studies are recommended.

## Note

***This article is extracted from student thesis (Fatemeh Shakarami). ***
